# A novel approach toward optimal workflow selection for DNA methylation biomarker discovery

**DOI:** 10.1186/s12859-024-05658-0

**Published:** 2024-01-23

**Authors:** Naghme Nazer, Mohammad Hossein Sepehri, Hoda Mohammadzade, Mahya Mehrmohamadi

**Affiliations:** 1 Department of Electrical Engineering, Sharif University of Technology, Tehran, Iran; 2 Department of Biotechnology, College of Science, University of Tehran, Tehran, Iran

**Keywords:** DNA methylation marker discovery, Simulation of DNA methylation array data, Data analysis pipeline optimization

## Abstract

DNA methylation is a major epigenetic modification involved in many physiological processes. Normal methylation patterns are disrupted in many diseases and methylation-based biomarkers have shown promise in several contexts. Marker discovery typically involves the analysis of publicly available DNA methylation data from high-throughput assays. Numerous methods for identification of differentially methylated biomarkers have been developed, making the need for best practices guidelines and context-specific analyses workflows exceedingly high. To this end, here we propose TASA, a novel method for simulating methylation array data in various scenarios. We then comprehensively assess different data analysis workflows using real and simulated data and suggest optimal start-to-finish analysis workflows. Our study demonstrates that the choice of analysis pipeline for DNA methylation-based marker discovery is crucial and different across different contexts.

**Supplementary Information:**

The online version contains supplementary material available at 10.1186/s12859-024-05658-0.

## Introduction

Re-analysis of publicly available high throughput data helps significantly improve the time and cost efficiency of research as well as allowing for large-scale integrative meta-analyses that are otherwise impossible. The bulk of publicly available DNA methylation data come from array or sequencing-based methylation profiling assays. In many disease contexts including cancer, a wealth of publicly available DNA methylation information exist from arrays including Illumina’s Human Methylation 450k and EPIC arrays. For instance, the cancer genome atlas (TCGA) [1] consists mostly of methylation arrays-based data which is widely used by cancer researchers for various applications (11,315 array-based vs. 585 sequencing-based). One of the most common applications of re-analyses of publicly available DNA methylation array data continues to be in marker identification. Variation in methylation level and pattern of certain positions in the genome are observed across different normal tissues as well as between healthy and diseased states of the same tissue. Such differentially methylated regions have been proven powerful as biomarkers for disease identification in a variety of contexts including cancer [2–4], autoimmune diseases [5–7], and neurodegenerative disorders [8].

Numerous computational tools have been developed for the identification of DNA methylation markers from array data. Minfi [9] and ChAMP [10] are among the most popular and comprehensive differential methylation analysis tools from R Bioconductor. Although these packages provide a flexible analysis method for differential methylation, they do not introduce a pipeline for the entire analysis process and are limited to the R programming environment. Recently, more general start-to-finish tools such as RnBeads [11], MADA [12], Ewastools (integrated into Galaxy) [13], and ADMIRE (Analysis of DNA Methylation In genomic Regions) [14] have helped overcome some of these challenges. However, these tools do not include best-practices guidelines for selection among the various parameters and options in each of the analysis steps. The choice of the right analysis method and processing steps depends on dataset characteristics and context and is not obvious in most instances.

Building an optimal analysis workflow by selecting a combination of tools based on input datasets and problems can be extremely challenging and calls for careful benchmarking efforts. Previous studies aiming to introduce such pipelines for the analysis of methylation data are often limited in scope. Some have only considered preprocessing (quality control, normalization and batch effect correction) steps in their comparisons [15–17] and others have focused on comparing across differential-methylation analysis algorithms [18]. Furthermore, for evaluation and comparison of different analysis methods, previous studies have mostly used array [12–14] or sequencing data [15, 16] in limited numbers and across a few contexts as the ground truth. Since the exact location of true differences between cases and controls is not accurately known beforehand, some studies have attempted to use matched methylation sequencing data as gold standard for true differentially methylated regions [15, 16]. However, this approach is also cost-prohibitive as well as sensitive to inaccuracies in sequencing-based methylation assays as previous studies have shown discrepancies between the two platforms, possibly due to both chip bias and sequencing bias [19]. Some comparisons even suggest array data outperform sequencing data in terms of precision [20], therefore, array data have themselves been used as gold standard for comparison of the performance of various pipelines of sequencing data analyses by others [21].

To overcome these limitations, simulated methylation data have been widely used for method comparison and benchmarking [18, 22–26]. This allows for accurate evaluation of performance measures such as precision and recall across methods. Starting from real methylation profiles, candidate differentially methylated regions (DMRs) can be chosen randomly [22, 23], using known regions from the literature, or by clustering methods [18, 24, 25]. After determining borders of DMRs to be simulated, a variety of approaches can be used for altering methylation levels and patterns in these selected regions. For instance, the methylation level of a single CpG site can be changed by adding a fixed value [18, 22] or a random variable from a beta distribution [23–25]. Some previous studies have also added a noise model to better reflect the technical and biological variabilities in real methylation data [22, 23]. Yet none of these studies have systematically quantified how well their simulation captures the technical and biological variations in real data, and have not evaluated their proposed pipelines under different contexts of dataset characteristics.

**Fig. 1:**
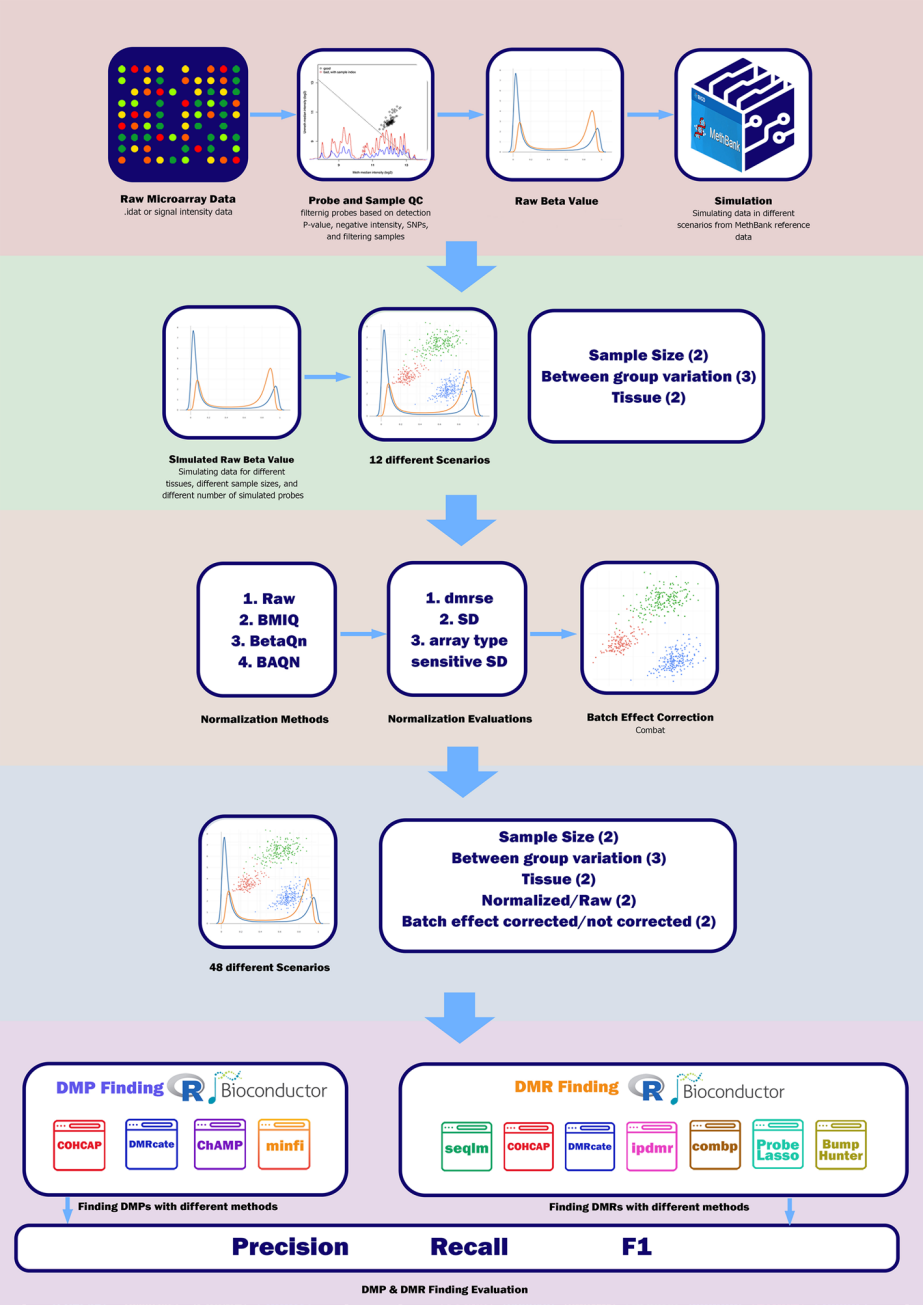
An overview of our benchmarking study. Publicly available methylation data on monocytes were obtained and put through quality control steps. The same data was used as input to a tissue-aware simulator (TASA). In total, 12 datasets were generated with different size and variations. Following normalization and batch effect correction, 48 different scenarios were input into DMP/DMR discovery tools. The best workflow was then presented for each of the 12 simulated datasets

Here, we propose a simulation method, TASA (Tissue Aware Simulation Approach), that uses reference methylation data to simulate known DMRs. This method accounts for biological and technical noise associated with real datasets while simulating certain regions with differential methylation. Next, 12 different contexts are simulated by TASA and for each, the most suitable combination of methods/tools are selected and a start-to-finish workflow is suggested (overview shown in Fig. 1).

## Methods

### Data quality control

Signal value of certain probes are prone to errors in methylation microarrays. It is common to identify and filter out such problematic probes so that they will not affect downstream analyses. Probes that fall in either of the following categories were excluded from our study: (1) probes with detection p-value more than 5%; (2) probes showing negative intensity value; (3) probes locating SNPs with an allele frequency of more than 5% (these probes may indicate occurrence of an SNP instead of site methylation.); (4) Non-specific probes, these probes may map to multiple locations in the genome [27, 28]. Low-quality samples were also removed from our analyses. Samples showing low median values both in M (methylated) and U (unmethylated) signal intensity were filtered out. For this, log2 transformed median value was calculated and values less than 10 in both M and U signals were removed.

To proceed, the beta-value was calculated from all downloaded datasets after quality control steps.

### TASA (tissue-aware simulation approach)

Adjacent CpG sites in the genome are known to be co-methylated [29]. Therefore, realistic methylation data simulators should take this correlation into account when artificially changing the value of methylation in a CpG site in the genome. To identify regions with high co-methylation across the genome, we used a clustering method and applied it to methylation data from 1202 samples of Monocytes (data accessible at NCBI GEO database [30], accession GSE56046 [31]). To simplify the analysis, only the first chromosome probes were considered. Depending on the location of the probe in the genome, probes were sorted. Pearson correlations were calculated for each probe across a window of size 3. To select regions exhibiting similar methylation values, probes showing an average correlation value more than a predefined threshold (0.1, 0.2, 0.4) were selected as candidates for simulating DMR.

Using the manufacturer-supplied annotation data (Infinium HumanMethylation450 v1.2 manifest file [32]), which contains information about the predicted length of HMM (Hidden Markov Model) islands in the genome, two thresholds were set to remove regions that were too far. One for regions with lengths below the minimum length of HMM islands (12 bases) and one for regions with two adjacent probes that were apart by more than 702 bases (the median length of HMM islands). This procedure yielded three sets of candidate regions for simulating DMRs. Each set represented a certain Pearson correlation threshold (0.1, 0.2, 0.4).

The beta-values were then simulated using reference datasets from Methbank for monocytes, breast tissue, and CD8 T-cells [33]. Methbank provides minimum, maximum, and average beta-values of each probe for each tissue. For tissue-aware simulation, we needed beta-values for all probes. The simulator used two probability distributions in series. (1) For each probe, a random selection of probability distributions was performed so that the selected value fell within the range of minimum and maximum beta-values of that probe for the target tissue in the Methbank dataset. It is repeated for the number of samples we aimed to simulate (n = 1202). After that, the average beta-values for each probe were used in the next step. (2) Using the average beta-value from the previous step and standard deviation from the source monocyte dataset (GSE56046 [31]), another distribution model was generated for each probe. A total of n = 1202 beta-value levels were then selected. The same process was carried out for all probes. Then, the average beta-value for each probe in the reference monocyte dataset from Methbank was subtracted from simulated beta-values. Finally, the residuals were added to the source monocyte data (GSE56046 [31]) and the final dataset was generated.

Four different simulation approaches were considered with different distributions and parameter settings. In the first simulation approach (S1), the source data of monocytes were simulated to target tissue by simply adding the difference between the average beta-value of each probe between Monocytes reference and CD8 T-cell reference data from Methbank to source Monocyte dataset (GSE56046 [31]). This is similar to the approach used by some previous studies [18].

$$Simulated\;Cell\;type\;Beta = Input\;Cell\;type\;Beta - \left( {\mu_{IR} - \mu_{SR} } \right)$$

In the second (S2) version, we used uniform probability distribution to calculate the average beta-value for each probe. Using that average value, another uniform distribution was used to reproduce n beta-values. The difference between these values and reference Monocyte data from Methbank was then added to the source Monocyte dataset (GSE56046 [31]).

$$min_{SR} \le b_{u} \le max_{SR}$$

$$Simulated\;Cell\;type\;Beta = Input\;Cell\;type\;Beta - \left( {\mu_{IR} - b_{u} } \right)$$

The third simulation (S3) is similar to the S2, while in S3 we used the uniform distribution followed by a normal distribution. This approach is somehow similar to other studies [25].

$$b_{i} = b\sim {\mathcal{N}}\left( {\mu_{SR} ,\sigma_{I} } \right)$$

$$Simulated\;Cell\;type\;Beta = Input\;Cell\;type\;Beta - \left( {\mu_{IR} - b_{i} } \right)$$

And in the fourth approach (S4), similar to the latter two approaches, two probability distributions were used in series. A beta distribution with alpha = 0.4 and beta = 0.5 was used to introduce within-region and between CpG variation, followed by a normal distribution for inter-sample variation [34].

$$a_{i} \sim \beta_{0.4,0.5} s.t.min_{SR} \le a_{i} \le max_{SR}$$

$$\overline{a} = \frac{1}{n}\mathop \sum \limits_{1}^{n} a_{i}$$

$$b_{i} \sim {\mathcal{N}}\left( {\overline{a},\sigma_{I} } \right)$$

$$Simulated\;Cell\;type\;Beta = Input\;Cell\;type\;Beta - \left( {\mu_{IR} - b_{i} } \right)$$

where ${a}_{i}$ has a beta distribution with alpha = 0.4 and beta = 0.5, and is between the minimum (${min}_{SR}$) and maximum (${max}_{SR}$) value of beta in MethBank's reference target tissue. $\overline{a}$ is the mean of n sample${a}_{i}$’s. ${b}_{i}$ is normal distribution with mean $\overline{a}$ and standard deviation ${\sigma }_{I}$ which is equal to the std of the source data. By adding the difference between the mean beta of the matching MethBank reference input cell type (${\mu }_{IR}$) and ${b}_{i}$ to the input beta-value of the source data, the simulated beta of each probe is obtained.

### Evaluation of TASA

Multiple Different distribution functions and parameters were used for TASA optimization. Cibersort cell-type deconvolution [35] and methylcibersort tool [36] were used to evaluate the outcome. This allowed us to compare our simulated dataset to real control datasets (accession GSE59065 [37]) in terms of cell-type deconvolution percentages. Also, PCA dispersion and an analysis of SVM classification were performed to evaluate how closely our simulated data matched the characteristics of a real dataset.

PCA dispersion was calculated in three manners. (1) the dispersion score between the simulated data and the matching reference control dataset (GSE59065 [37]). Simulator performance is better when this score is lower. (2) the dispersion score between the simulated data and the reference data of the source tissue (GSE103541 [38]). In this case, a larger amount is better. (3) The dispersion score between the group of simulated data and the matching reference dataset (GSE59065 [37]) vs. the group of control data and the reference dataset of the source tissue (GSE56046 [31], GSE103541 [38]). A higher score shows better performance here as well.

The SVM algorithm was trained and tested using real datasets of the same tissues (monocytes as our source tissue, CD8 T cells, and breasts as our target tissues). We obtained 138 samples of monocyte (accession GSE56046 [31] and GSE103541 [38]), 128 samples of CD8 T cells (accession GSE103541 [38] and GSE59065 [37]), and 121 samples of breast tissue (accession GSE101961 [39]) from GEO. We randomly selected 80% of the data for training. PCA was trained using the training data and then was used to reduce the dimension of these data. Afterwards, the first ten PCs were used to train the SVM classifier with tenfold cross-validation and 100% accuracy was obtained on the training data. Then, test dataset was projected onto the trained PCA space and was labeled using the trained SVM resulting in 100% accuracy. Then, we analyzed our simulated data consisting of three tissues using PCA transformation and the SVM classifier trained on our training set to see if they can be classified correctly. The average of absolute decision values were used to choose the best simulation method.

### Normalization

Considering that our simulation is based on beta-values, we chose normalization techniques that are applicable to beta-values. BetaQN [40], BMIQ [40, 41] and Between Array Quantile Normalization (BAQN) were applied and compared against raw unnormalized beta-values based on defined evaluation metrics explained in the following. In BAQN, probes belonging to type I or type II were separately quantile normalized.

To compare normalization techniques, four evaluation metrics were calculated: (1) Median of probe SDs (Standard Deviations) across samples, (2) Median of type-1 probe SDs across samples, (3) Median of type-2 probe SDs across samples, and (4) dmrse (differentially methylated region standard error) [40]. In each simulation scenario, the output that outperformed the others according to these evaluation criteria was chosen (Additional file 1: Table S1). For the rest of our benchmark, we just compared BAQN with raw data since BAQN showed superior performance in all scenarios.

### Batch effect correction

Initially, five different datasets of monocyte tissue were used to simulate different batches (GSE56046 [31], GSE120610 [42], GSE131989 [43], GSE134429 [44], and GSE184269 [45]). The small and large size datasets were constructed by selecting samples from each dataset (Table 1). Afterward, half of the samples from each dataset were simulated into the target tissue, while the other half remained as monocytes. Using combat [46], batch effect correction was performed for each scenario and stored for comparison to determine whether or not batch effect had to be corrected.

**Table 1:** Number of samples selected from each monocyte dataset for small and large size data scenarios

Input methylation datasets for simulation	Number of samples selected for large size scenario	Number of samples selected for small size scenario
GSE56046	204	14
GSE120610	156	6
GSE131989	31	4
GSE134429	17	4
GSE184269	24	4
Sum	432	32

### Evaluation of DMP finding methods

DMPs can be found using a variety of methods and packages. Most of them follow the statistical algorithms and parameters of regular ANOVA. For benchmarking DMP finding methods in different scenarios, we selected four of the most commonly used. The tools were minfi [9], ChAMP [10], COHCAP [47], and DMRcate [23, 48]. All were run with their default settings.

For each DMP detection method, we first calculated TP (True Positive), TN (True Negative), FP (False Positive), and FN (False Negative). A comparison between the simulated and predicted DMPs was conducted for this purpose. Then, we calculated sensitivity, specificity, accuracy, precision, recall, and F1-score, and then used the F1 score as the final determining factor.

### Evaluation of DMR finding methods

Many tools have been developed and are available for finding significant DMRs between two groups. Here seven techniques which are amongst the most popular methods were selected for benchmarking. These include BumpHunter [49], ProbeLasso [50], seqlm [25], DMRcate [23], COHCAP [47], Comb-p [51], and ipDMR [52]. All of them were used with their default parameter settings.

Precision, recall, and F1 scores for detecting simulated differential methylation signal were calculated for each scenario to determine the overall pipeline performance. To accomplish this, it is necessary to define TP, FP, and FN. To do so, each detection was compared against 20% overlap criteria as follows. For each simulated region, if the pipeline found a simulated region with an overlap of more than 20% with the true simulation boundaries, this was a TP, whereas if it was covered by less than 20% with a pipeline-detected region, it was a FN. If the pipeline detected a new region with less than 20% overlap with any simulated region, then it was considered a FP.

### Usage guide

This guideline outlines the steps for choosing the optimal workflow for analysis of DNA methylation array data. Here, we have conducting methylation data analysis using an example dataset (GSE87053) [53], which comprises of 21 samples of oral squamous cell carcinoma (OSCC) disease and adjacent normal tissue.

*Step 1: Data retrieval and initial preparation*

1. Download “GSE87053_RAW.tar” and “GSE87053_series_matrix.txt.gz” from the Gene Expression Omnibus (GEO) using accession number GSE87053. The original dataset includes 485,512 probes and 21 samples.
2. Make three directories. 1- “GSE87053”, 2- “code”, 3- “info_datasets”.
3. In directory “GSE87053”, make another directory and name it “idat”. Place the two downloaded datasets in the “GSE87053” folder. Download the “confirmed_genes.csv” from link below and place it in the “GSE87053” folder. https://github.com/NaghmeNazer/TASA-benchmerk/tree/main/example
4. Download “test_data_beta_generate.R”, “test_data_DMRcate.R”, “test_data_DMR.R” from github link and place them in the “code” folder. https://github.com/NaghmeNazer/TASA-benchmerk/tree/main/example
5. Download “rs_af0.05.csv”, “non_specific_sites.csv” from github link and put them in the “info_datastes” folder. https://github.com/NaghmeNazer/TASA-benchmerk/tree/main/example
6. Download “450k_manifest.csv” from link below and place it to the “info_datasets” folder. https://drive.google.com/file/d/11U4kpdnaGZGlS8aOGOlm_-8X7ZUX64K8/view?usp=share_link

*Step 2: Quality Control, Data Preprocessing and Beta Value Generation*

1. Open “test_data_beta_generate.R”. This script reads the raw data, filters the bad quality probes and samples, and finally generates beta value matrix and write it to the “GSE87053” folder.
2. The code loads the required libraries. (Lines 1 to 5)
3. There are functions for preprocessing and beta value generation. (Lines 7 to 77)
4. It reads the dataset and makes the M and U matrices containing methylated and unmethylated signal intensities for all probes and samples. To do so it uses the minfi package. (Lines 79 to 93)
5. It does the preprocessing based on M and U matrices and calculates the beta value for remained probes and samples. Beta values are stored in B matrix and written to the “GSE87053” folder. After filtering, the data will contain 401,896 probes and 21 samples. (Lines 94 to 98)
6. The next section performs PCA on the data and generates the PCA plot of first two PCs. The result plot will show us how much the tissues in the comparison are different. Based on this plot we can see that the two groups (Normal and OSCC) are not that much different as we also expected. The PCA plot will be saved in the “GSE87053” directory. (Lines 100 to 113)

*Step 3: Workflow Selection*

1. Given the nature of the dataset, where differences of small effect size are expected between the two groups (tumor vs. adjacent normal), and also what we observed in the PCA analysis, we select the "Small Size Data (< 50)/Small Difference Tissue" workflow.
2. The manuscript recommended pipeline includes no normalization but batch effects coming from using different datasets must get corrected. Batch effect correction step is skipped given the dataset consists only of one batch. According to the pipeline, DMRcate is used for the identification of Differential Methylation Probes (DMPs) and ipdmr for Differential Methylated Regions (DMRs).

*Step 4: DMP Identification*

1. Run “test_data_DMRcate.R”
2. The code loads the required libraries. (Lines 1 to 3)
3. It reads the beta value matrix generated in the Step 2. (Lines 5 to 10)
4. Using the series matrix, it gets the phenotype (Normal vs OSCC) of each sample. (Lines 12 to 17)
5. Finaly it uses DMRcate to find the DMPs between two groups. It will save the resulting DMPs in the “GSE87053” folder. It finds 67,973 DMPs. (Lines 19 to 24)

*Step 5: DMR Identification*

1. Run “test_data_DMR.R”. This code finds the DMRs with ipdmr method as pipeline suggested. Then to evaluate the results of the suggested pipeline, the code intersects the resulting DMR list with a list of independently confirmed genes by authors.
2. It loads the required libraries. (Lines 1 to 5)
3. It reads the manifest file of 450K array. This file will be used to annotate the probes to genomic locations. (Lines 7 to 9)
4. It reads the beta values generated in Step 2. (Lines 11 to 16)
5. It identifies the group of each sample using the series matrix. (Lines 18 to 22)
6. To run ipDMR, we need the identified DMPs. We use the DMPs detected in the Step 4. (Lines 24 to 31)
7. Then ipdmr is used to identify the DMRs. It returns 44,121 DMRS, from which 10,346 DMRs contain more than one probe (Lines 33 to 38)
8. It reads the list of confirmed genes and make it to the format of a bed file. (Lines 40 to 42)
9. It reformats the result of ipdmr to a bed file format. (Lines 44 to 48)
10. It intersects the ipdmr results with confirmed genes. We see that 12 out of 14 genes are identified using this pipeline. (Lines 50 to 54)

## Results

### Tissue-aware simulation mimics real methylation data

Given the limitations of the existing simulation tools for DNA methylation array data and the importance of this step in benchmarking as discussed above, we first developed a novel simulator. The goal of this tissue-aware simulation approach (TASA) was to: (1) generate *in-silico* array data that closely mimic real data, and (2) simulate case and control datasets under a variety of contexts in terms of scale and variation in the case and control cohorts.

**Fig. 2:**
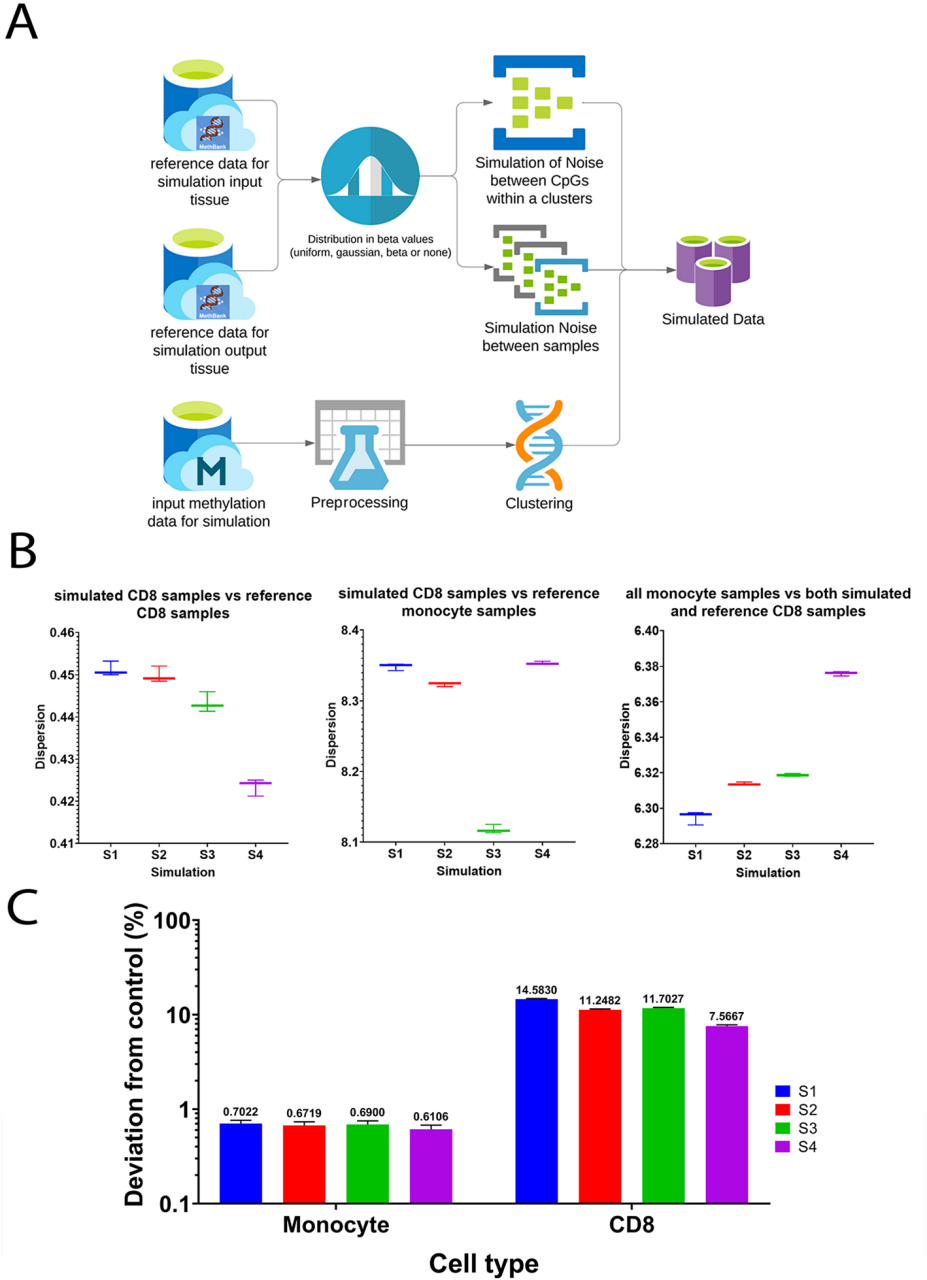
The overall design of the tissue-aware simulator (TASA) and the evaluation methods to select the best approach. **A **Simulation starts from a source tissue data and a target tissue of interest. For identifying candidate DMR region borders, source data is preprocessed and clustered. For both the source (input) and target tissues (output), the reference data from Methbank [33] were used. Two distributions were sampled to mimic the characteristics of the output tissues, and the differences in methylation level between the outputs and inputs were added to the beta-values of probes in the candidate DMR regions. Using this algorithm, 4 different approaches were considered to simulate the target tissues. **B** Scores of PCA dispersion in four different simulation approaches. Graph 1 shows the dispersion score between simulated and real CD8 samples (GSE59065 [37]), lowest score is the best. Second, the dispersion score between simulated CD8 samples and real Monocyte samples (GSE103541) is shown, with a higher score indicating better performance. Third shows the dispersion score between real monocytes (GSE56046[31], GSE103541 [38]), and a combination of real (GSE59065 [37]) and simulated CD8 samples. A higher score indicates a better performance. **C** Comparison of cell-type deconvolution percentages between simulated data and matched control dataset on a logarithmic scale. Cell-type percentages were calculated for different CD8 simulation approaches and for a real CD8 control dataset (GSE59065 [37]). Different datasets are represented by different colors

Starting with a given dataset of array methylations across two groups (i.e. source and target tissue types), our simulator identifies boundaries of co-methylated regions to be simulated using an unbiased clustering approach in the target tissue (see Methods). Next, CpG methylation levels (beta-values) in the source group are altered by sampling from two probability distributions. First a distribution for variation in methylation across CpGs within a given co-methylated region, and then a second distribution for variation in the methylation level of the same CpG across different individual samples (Fig. 2A). Here, we used monocyte methylation profiles from five different datasets (GSE56046 [31], GSE120610 [42], GSE131989 [43], GSE134429 [44], and GSE184269 [45]), and simulated CD8 and breast as our target tissues using reference methylation data from Methbank [33].

We evaluated four different parameter settings (S1 to S4) for TASA to optimize our simulator using monocyte, breast and CD8 cell methylation profiles as reference. In the S1 approach, we simply simulated the target tissue by adding the difference between the average beta-values of target and source tissues in the Methbank dataset to the source monocyte dataset. The other three approaches used two probability distributions to generate the beta-values of the target tissue. The difference between these beta-values and the mean beta-value from reference Methbank data was then added to the source monocyte database. In S2, we used two uniform distributions while in S3, we used a uniform distribution across CpGs within a sample followed by a normal distribution for each CpG across different simulated samples. Finally, in the fourth setting (S4) we used a beta distribution with alpha = 0.4 and beta = 0.5 followed by normal distribution (see Methods).

**Fig. 3:**
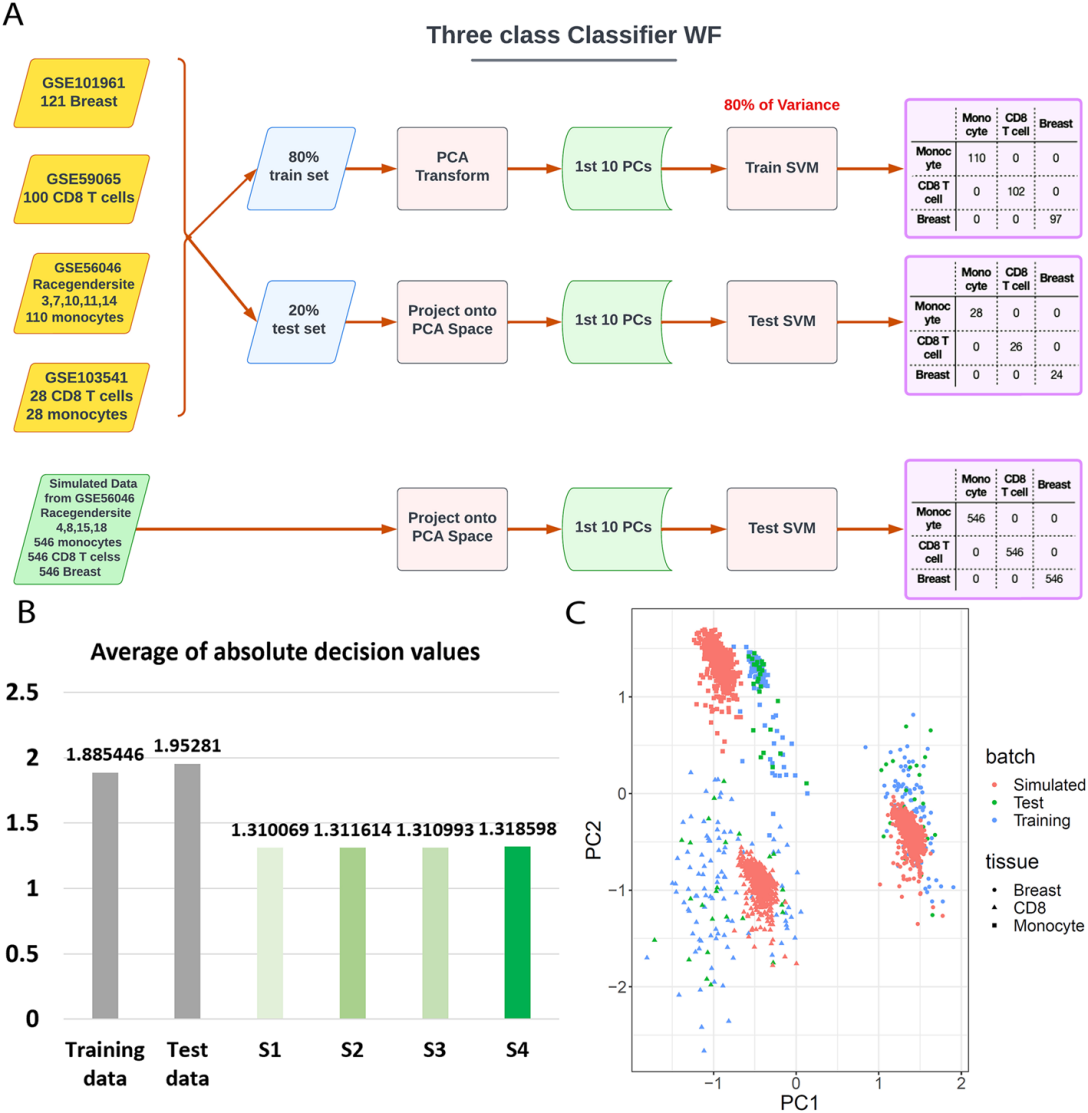
The SVM classifier used for evaluating TASA. **A** Schematic summary of the classification scheme. Training and test samples were obtained from GEO on CD8, Breast, and Monocyte datasets. Training was conducted on the first 10 PCs of the training dataset (random 80% of samples) and testing was conducted on the test dataset (remaining 20%). Next, the trained model was used to classify simulated samples. **B** The average of absolute decision values from the SVM classifier is used as an evaluation score for each simulation approach. Each simulation approach is color-coded according to its score. The higher the score, the better the simulation. **C** PCA plot of the simulated and real datasets. As the best performer of all approaches, S4 generated the simulated samples shown in this panel. Tissue attributes are represented by the shape of dots, and batches with colors (red for simulated data, green for test data, and blue for train data)

Next, we compared the above four parameter settings to choose the best one for TASA assuming the desired simulator is the one that best mimics the characteristics and natural variability of the target tissues. To this end, three evaluation tests were performed: (1) Cell-type deconvolution, (2) PCA dispersion, and (3) SVM classification. The cell-type deconvolution test was performed only when CD8 was used as the target tissue. The simulated datasets were decomposed into cell types of origin and compared against a real control dataset of pure CD8 cells (GSE59065[37]). The results across the four methods were similar, however, S4 had the closest estimated cell fractions to the real control dataset (Fig. 2B, see Methods). A PCA dispersion evaluation was conducted using three metrics as measures of similarity/difference between simulated samples and target tissues: (1) Dispersion score between simulated and real samples of the matching target tissue (GSE59065[37]); (2) Dispersion score between simulated and real samples of the source dataset (GSE103541[38]); and (3) Dispersion score between the group of simulated and real samples of matching target tissue (GSE59065[37]), and real samples of the source dataset (GSE56046[31], GSE103541[38]). In all three evaluations, S4 showed the best performance (Fig. 2C). Finally, a 3-class SVM classifier was trained and tested on samples of matching tissues of monocytes, CD8 and breast (GSE56046 [31], GSE103541 [38], GSE59065 [37], GSE101961 [39]) (Fig. 3A). Data simulated by all of the four simulation approaches were correctly classified using the trained SVM (see Methods). Comparing simulated datasets using the average of absolute decision values, we observed that S4 was the best performer again (Fig. 3B, see Methods). The PCA plot of the samples shows similarities between the simulated datasets and the tissues they represented (Fig. 3C). Overall, we decided to use S4 parameters in the core of TASA for all future simulations.

Next, the following two strategies were used to create datasets with varying levels of methylation difference between the case and control groups: (1) Simulation of two different target tissues (breast and CD8) from the source tissue (monocyte). The average absolute difference in methylation level between target and source probes in each cluster was computed, and histogram density of these values revealed more variable regions in breast tissue than CD8 cells. Hence, TASA produced a bigger change amplitude when attempting to simulate breast tissue vs, CD8 cells (Additional file 6: Fig. 1); and (2) The use of different correlation thresholds (0.1, 0.2, 0.4) in the clustering step (see Methods). At lower correlation thresholds, more probes were selected for simulation, resulting in more diverse groups. Control datasets without DMR simulations were also generated, dividing the samples into two groups of reference (monocytes) and target tissues (CD8/breast), but with no change in the probes. Finally, in each context, two different dataset sizes were simulated: small (n = 32 samples) and large (n = 432 samples) to test the effect of sample size.

### Identifying optimal DMP finding workflows

With our finalized TASA tool (S4), we next simulated methylation array datasets under a variety of contexts. Absolute differences in methylation between case and control groups were simulated either by altering the magnitude of difference in beta-values between source and target tissues (large vs. small), or by altering the number of differentially methylated sites (small, medium, large). For instance, the former is obtained by simulating to two very distinct target tissues (CD8 and Breast), while the latter is achieved by altering the methylation levels in different numbers of probes, resulting in different number of altered probes between the two groups. In addition to this criteria, we also considered variable sample sizes (small vs. large) in all datasets generated. This resulted in 12 benchmark scenarios that were evaluated in different stages of the analysis.

**Fig. 4:**
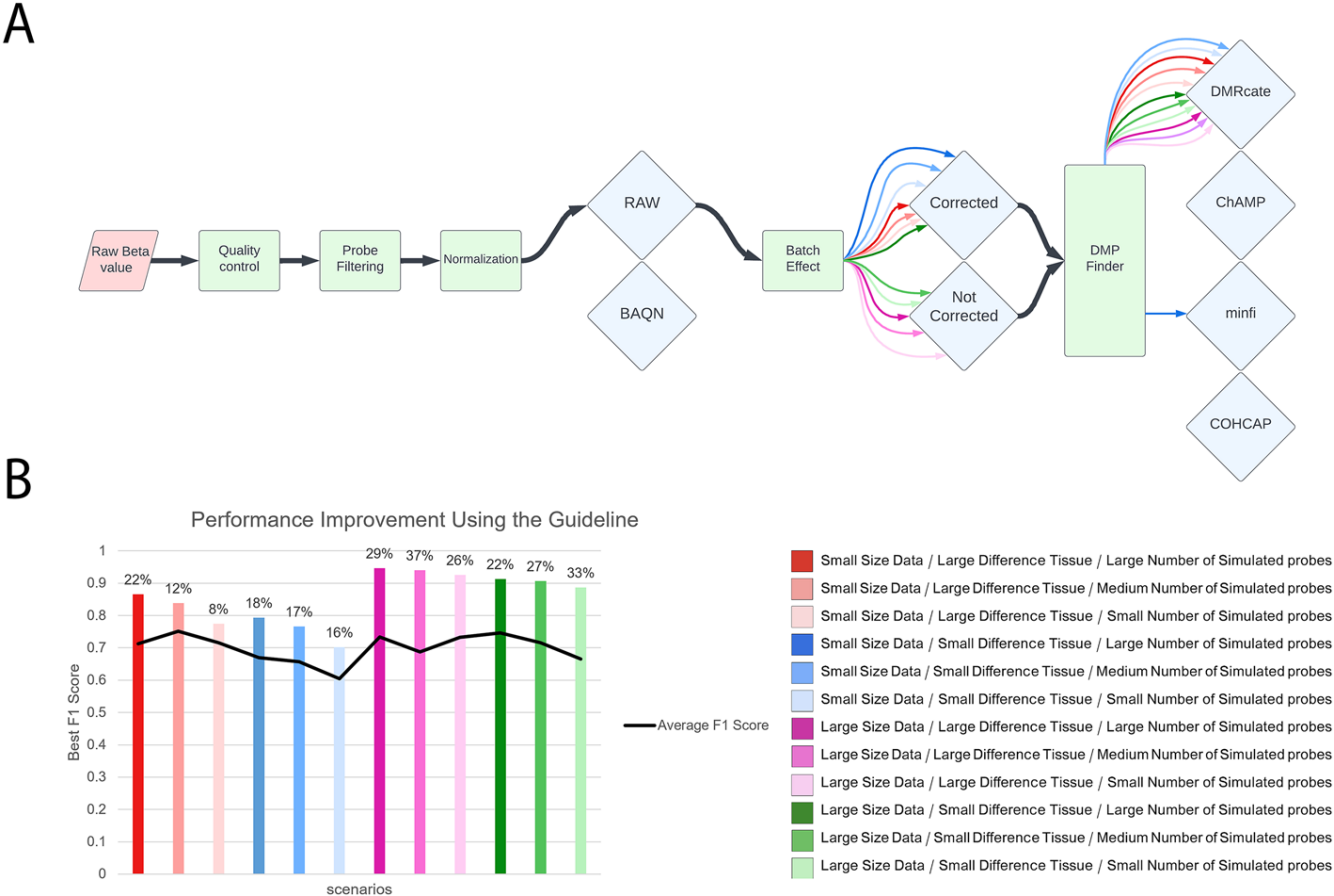
Guideline for selecting the best combination of steps towards DMP finding based on the F1 score. Twelve simulation scenarios were developed that varied in sample size (small or large), simulation target tissue (breast-large tissue difference or CD8-small tissue difference), and the number of altered probes (small, medium, or large). **A** Normalization, batch effect correction, and four DMP finding tools were considered. The different input characteristics are represented by color and for each of them, the best pipeline can be selected by looking at the diagram. **B** In each scenario, the F1-score improves when using the guideline compared to the average F1-score of all possible pipelines. In each input scenario, the bar label indicates the enhancement percentage

There are three key steps in common analysis pipelines for DNA methylation data: normalization, batch effect correction, and DMP/DMR identification. Here, for each of the 12 contexts described above, our simulated markers were used as ground truth and different evaluation metrics such as precision, recall, accuracy, and F1-score were calculated to select an optimal pipeline (Additional file 2: Table S2). Based on the F1 score, we identified the best start-to-finish DMP finding pipelines for 12 scenarios (Fig. 4A). As compared to the average F1-score from all pipelines possible for the specific input scenario, using our guideline to select the best combination will improve the F1-score in 12 simulation scenarios by 22.21% (Fig. 4B). Our results suggest that normalizing is unnecessary and batch effect correction is only beneficial for small datasets (N < 50). In each scenario, the four DMP finding methods have performed almost similarly, and there was no significant difference between them. However, in contexts with small differences between cases and controls, DMRcate performed better in the majority of scenarios (Fig. 4B).

### Identifying optimal DMR finding workflows

**Fig. 5:**
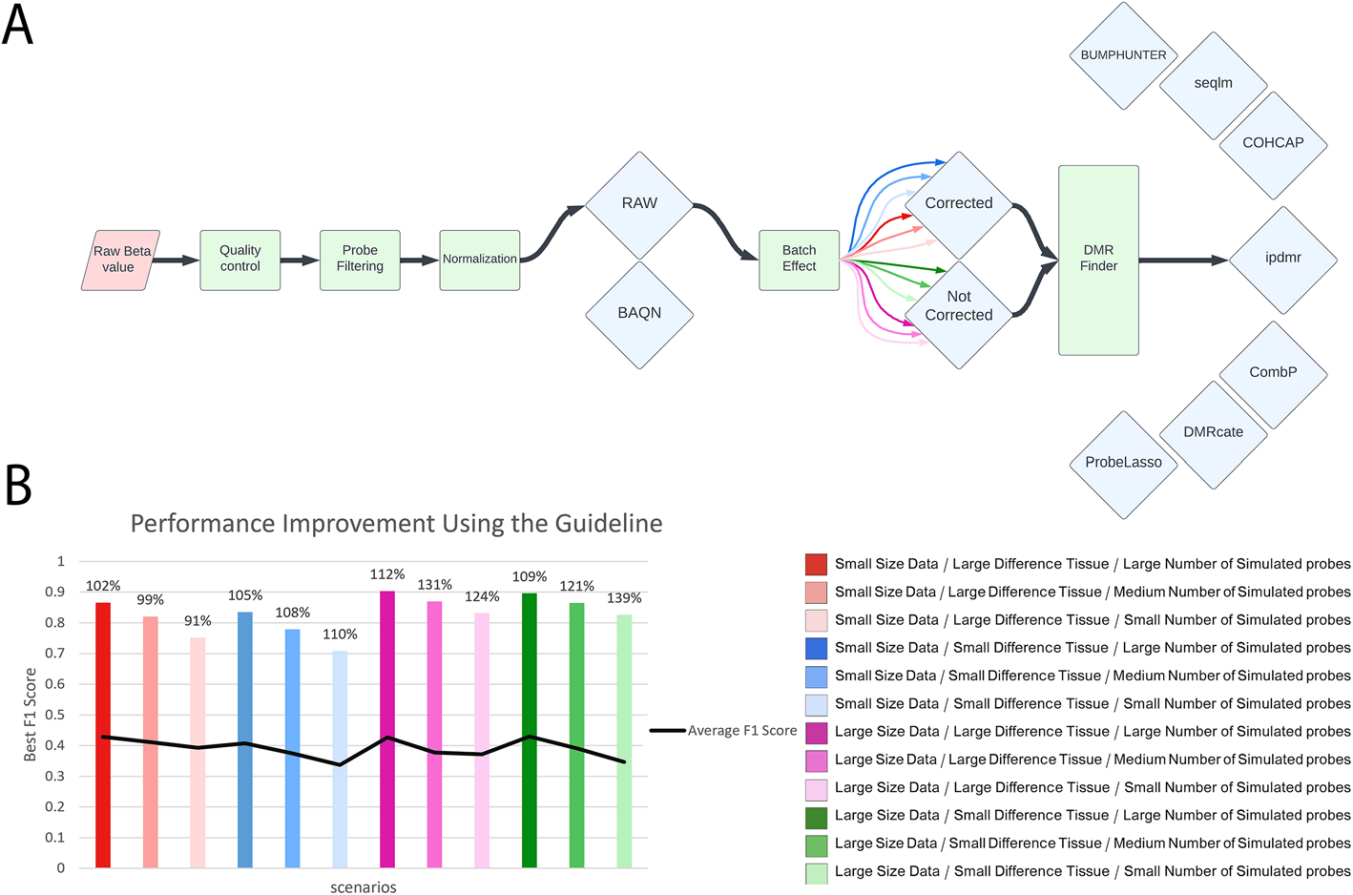
Guideline for selecting the best combination of steps towards DMR finding based on the F1 score. Twelve simulation scenarios were developed that varied in sample size (small or large), simulation target tissue (breast-large tissue difference or CD8-small tissue difference), and the number of altered probes (small, medium, or large). **A** Normalization, batch effect correction, and seven DMR finding tools were considered. The different input characteristics are represented by color and for each of them, the best pipeline can be selected by looking at the diagram. **B** In each scenario, the F1-score improves when using the guideline compared to the average F1-score of all possible pipelines. In each input scenario, the bar label indicates the enhancement percentage

To identify optimal workflows for finding DMRs, we similarly compared F1 scores across various workflows (Additional file 3: Table S3). The method ipDMR [52] consistently outperformed other DMR finding methods, according to the comparison analyses (Fig. 5A). Normalization seemed unnecessary in every scenario, and batch effect removal was helpful for small datasets. Overall, our guidelines improved the F1-score in 12 simulation scenarios by 112.58% compared to the average F1-score from all pipelines (Fig. 5B).

It is important to note that the F1 was the basis of these results. A preference for fewer false positive occurrences, for example, can make precision the preferred metric to optimize in certain contexts. We observed that ProbeLasso performs better based on precision (Additional file 3: Table S3).

Differentially methylated regions in a biomarker discovery study differ in the amplitude of methylation difference between the case and control groups (i.e. fold change). It is often more interesting to identify fewer DMRs but with a larger difference between comparison groups. In order to find the optimal pipeline specifically with regards to fold-change, we calculated the average change in the probes of each simulated region. Then with applying the threshold of 0.3, regions with higher fold changes (average beta value difference in the DMR > 0.3) were separated from the rest (average beta value difference in the DMR < 0.3). We then applied all the above steps separately to these two sets to assess whether the conclusions differ significantly. Then, the optimal pipelines for detecting specific DMRs with large/small fold change were determined (Additional file 4: Table S4, Additional file 6: Figure S2, Additional file 5: Table S5, Additional file 6: Figure S3).

### Validation

To demonstrate the effectiveness of our guideline in producing reliable results, we conducted a comparative analysis. We used an independent dataset (accession GSE8753) and analyzed it with the recommended workflow outlined in our guideline. We then contrasted the outcomes of our approach with a commonly used analysis pipeline. All of the preprocessing steps were kept consistent between the two pipelines, and only the DMR finding algorithm used was different. Subsequently, we evaluated the overlap between the regions identified by ‘ipdmr’ in our pipeline vs. ‘bumphunter’ in the alternative pipeline. The authors in the original study reported a list of 14 genes identified as differentially methylated through the analysis of their 450k dataset and further confirmed via independent validation using quantitative real-time methylation-specific PCR. Results from our pipeline overlapped with 12 out of the 14 genes, while the alternative pipeline failed to detect any of them (Additional file 6: Figure S4). This analysis further demonstrated that our proposed guidelines are useful for achieving reliable results.

## Conclusions

At the forefront of biomarker discovery, high -throughput technologies such as Next-Generation Sequencing (NGS) and Methylation Arrays (450K, EPIC) have revolutionized our comprehension of DNA methylation. This duo combines the efficiency and cost-effectiveness of arrays, offering high-throughput profiling, with the unparalleled resolution provided by NGS. As a result, the vast data available on methylation can be effectively employed, using statistical and machine learning methods, to discover biomarkers in different settings. Together, they have identified potential biomarkers with profound implications for medicine spanning early disease detection, prognosis, and the identification of treatment targets.

However, while this technological synergy has significantly advanced biomarker discovery, challenges persist in ensuring the consistency and reproducibility of biomarkers when tested across different batches, laboratory settings, and demographic cohorts. Addressing this challenge, our approach involves the development of a simulator that utilizes two distributions to model both inter-sample and within-sample variations.

This systematic approach establishes a best practice for biomarker discovery across diverse scenarios, aiming for robust and reliable results.

Methylation array data analysis consists of multiple steps, and choosing the appropriate combination of parameters, tools, or options given the analysis context can be challenging. Hence, comprehensive benchmarking efforts to suggest best practices guidelines are needed for various study contexts. Simulated data facilitate this task efficiently with minimal costs. However, between-sample and within-sample variations in simulated data typically deviate from those of real data. Statistical tests for the identification of differentially methylated markers between cases and controls are significantly impacted by the noise levels present in the data. Therefore, it is crucial to simulate methylation data that closely mimic true biological variation. Here, we developed a simulation approach, TASA, to generate realistic datasets differing in sample size and between-group variation in methylation levels. TASA, our proposed tissue-aware simulation approach, offers a significant advancement in the realm of methylation array data simulation for research and benchmarking purposes. This innovative simulator holds several key advantages that make it a valuable tool in the field. TASA excels in generating in-silico array data that closely mirror real-world data, capturing the nuances of both biological and technical variation present in actual datasets. Its flexibility allows for the generation of simulated datasets under a variety of contexts, making it adaptable to diverse study scenarios. Furthermore, our extensive evaluation indicates that TASA, particularly when configured with S4 parameters, consistently outperforms other simulation methods in closely mimicking the characteristics and natural variability of target tissues, as demonstrated by successful cell-type deconvolution, PCA dispersion, and SVM classification tests. However, it is essential to acknowledge certain limitations of TASA. While it effectively addresses many simulation challenges, it may not capture the full complexity of next-generation sequencing-based data, which require a distinct approach. Additionally, TASA's performance is closely tied to the quality and representativeness of the reference datasets, necessitating thoughtful consideration when selecting source and target tissues. Nevertheless, the advantages of TASA in generating realistic, context-specific simulated data for methylation array analysis are evident and promise to enhance the efficiency and accuracy of research in the field.

Compared to previous benchmarking studies, our study is more comprehensive with respect to both the scope of the workflow optimization (pre-processing to marker selection) as well as the diversity of the contexts considered (12 different case–control datasets). In this study, DMP/DMR finding tools were used with their default settings and then compared based on F1 scores. Our results suggested that normalization (by BAQN; see Methods) is not beneficial while batch effect correction can be beneficial when analyzing small datasets (< 50), but it seems unnecessary if the datasets are large (> 400). These conclusions may change by changing source data used as input for TASA and should thus be interpreted with caution. The best-performing tools for DMP and DMR findings were DMRCate [23] and ipdmr [52] in our analyses. In order to simplify the analysis, the benchmark was run with default parameter settings for each of the above tools, but one can investigate the parameters further to gain more insight in the future.

Although this manuscript was focused on methylation microarray data, the insights and findings presented here are extendable in many contexts to methylation data from next-generation sequencing-based assays as well. More specifically, the post-processing including DMP/DMR finding steps are commonly used for marker identification from sequencing data after converting methylation calls to beta-values. The read-level methylation and co-methylation information from NGS-based assays however, are unique to this type of data and thus analysis steps and algorithms that leverage such methylation haplotype information [54] were not included in our study. In the future, TASA can be extended to simulate read-level methylation data, followed by benchmarking approaches to identify best practices workflows.

### Supplementary Information

**Additional file 1**. Table S1. Comparison of different normalization techniques (BMIQ, betaQN, BAQN) on all simulation. scenarios

**Additional file 2**. Table S2. Results from evaluating different pipelines in analyzing all simulation scenarios for DMP finding.

**Additional file 3**. Table S3. Results from evaluating different pipelines in analyzing all simulation scenarios for DMR finding.

**Additional file 4**. Table S4. Results from evaluating different pipelines in analyzing all simulation scenarios for DMR finding in detecting regions with small difference.

**Additional file 5**. Table S5, Results from evaluating different pipelines in analyzing all simulation scenarios for DMR finding in detecting regions with large difference.

**Additional file 6**. Figures S1–S4.

## Data Availability

The datasets supporting the conclusions of this article are available in NCBI Gene Expression Omnibus, accession numbers: GSE56046, GSE59065, GSE103541, GSE101961, GSE120610, GSE131989, GSE134429, and GSE184269. R code generated for the analyses in this study is available at https://github.com/NaghmeNazer/TASA-benchmerk.
