# Supplementary material for: A novel approach toward optimal workflow selection for DNA methylation biomarker discovery
Source: BMC Bioinformatics. 2024 Jan 23;25:37. doi: 10.1186/s12859-024-05658-0 (PMC10804576; doi:10.1186/s12859-024-05658-0)
Supplement: Supplementary file 6 — Additional file 6. Figures S1–S4. [file 12859_2024_5658_MOESM6_ESM.docx]

**Supplementary Figure 1**


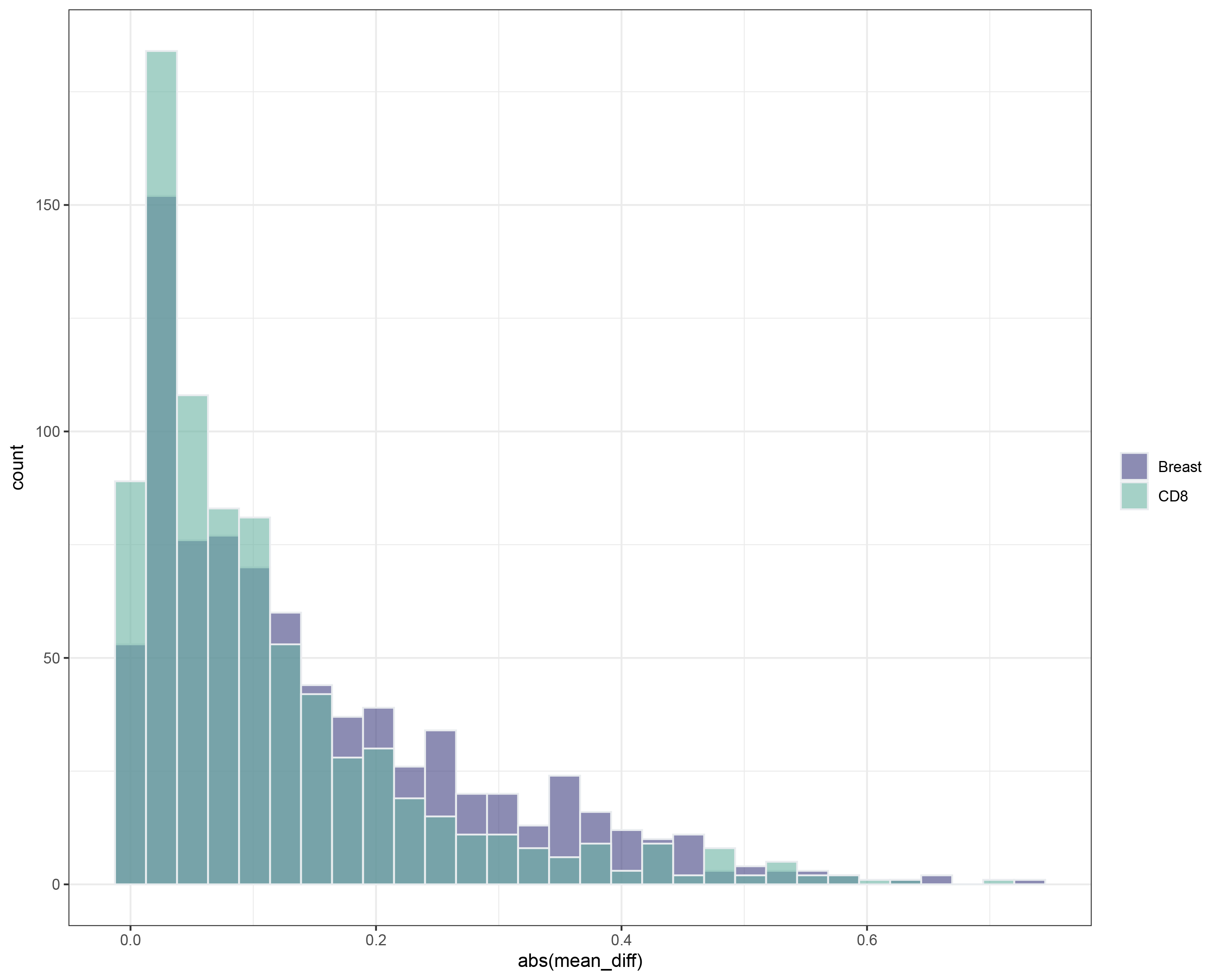


*Histogram density of the absolute value of average differences between the target (CD8 or Breast) and the source tissue (Monocyte) in each cluster. CD8 is represented with green and breast tissue with purple. Compared to Monocytes, Breast tissue has a more distinctive methylation profile than CD8 tissue based on the distribution.*

**Supplementary Figure 2**

**
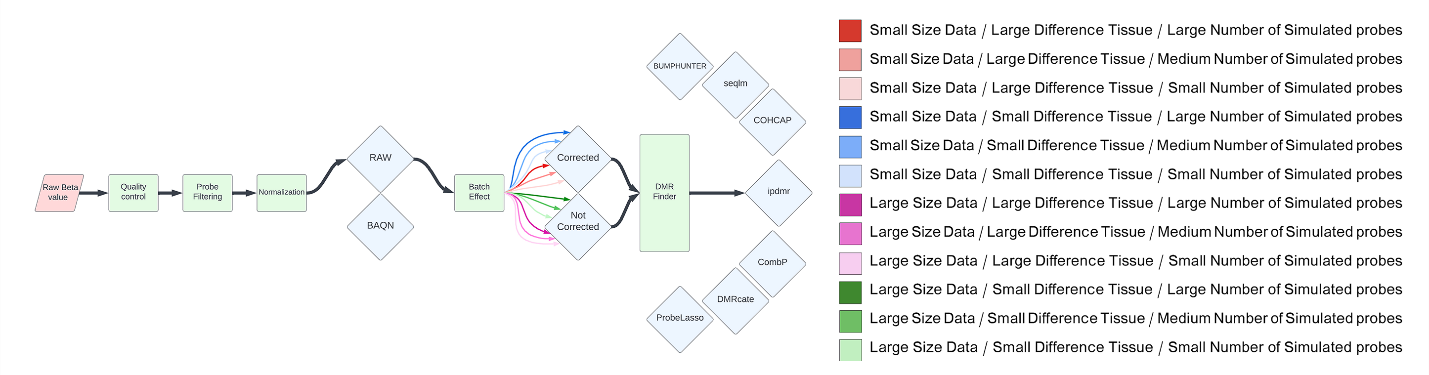
**

*Guideline for selecting the best combination of steps based on the F1 score. Twelve simulation scenarios were developed that varied in sample size (small or large), simulation target tissue (breast-large tissue difference or CD8-small tissue difference), and the number of altered probes (small, medium, or large). The guideline shows the best pipeline for finding DMRs between two groups when just looking at low differentiated DMR clusters. normalization, batch effect correction, and 7 DMR finding tools were considered. The different input characteristics are represented by color and for each of them, the best pipeline can be selected by looking at the diagram.*

**Supplementary Figure 3**

**
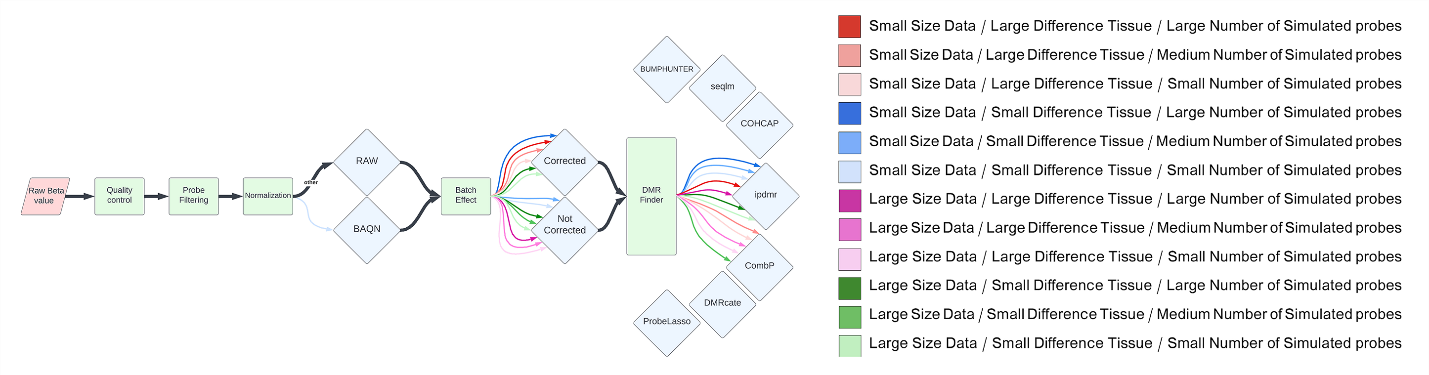
**

*Guideline for selecting the best combination of steps based on the F1 score. Twelve simulation scenarios were developed that varied in sample size (small or large), simulation target tissue (breast-large tissue difference or CD8-small tissue difference), and the number of altered probes (small, medium, or large). The guideline shows the best pipeline for finding DMRs between two groups when just looking at high differentiated DMR clusters. normalization, batch effect correction, and 7 DMR finding tools were considered. The different input characteristics are represented by color and for each of them, the best pipeline can be selected by looking at the diagram.*

**Supplementary Figure 4**

**
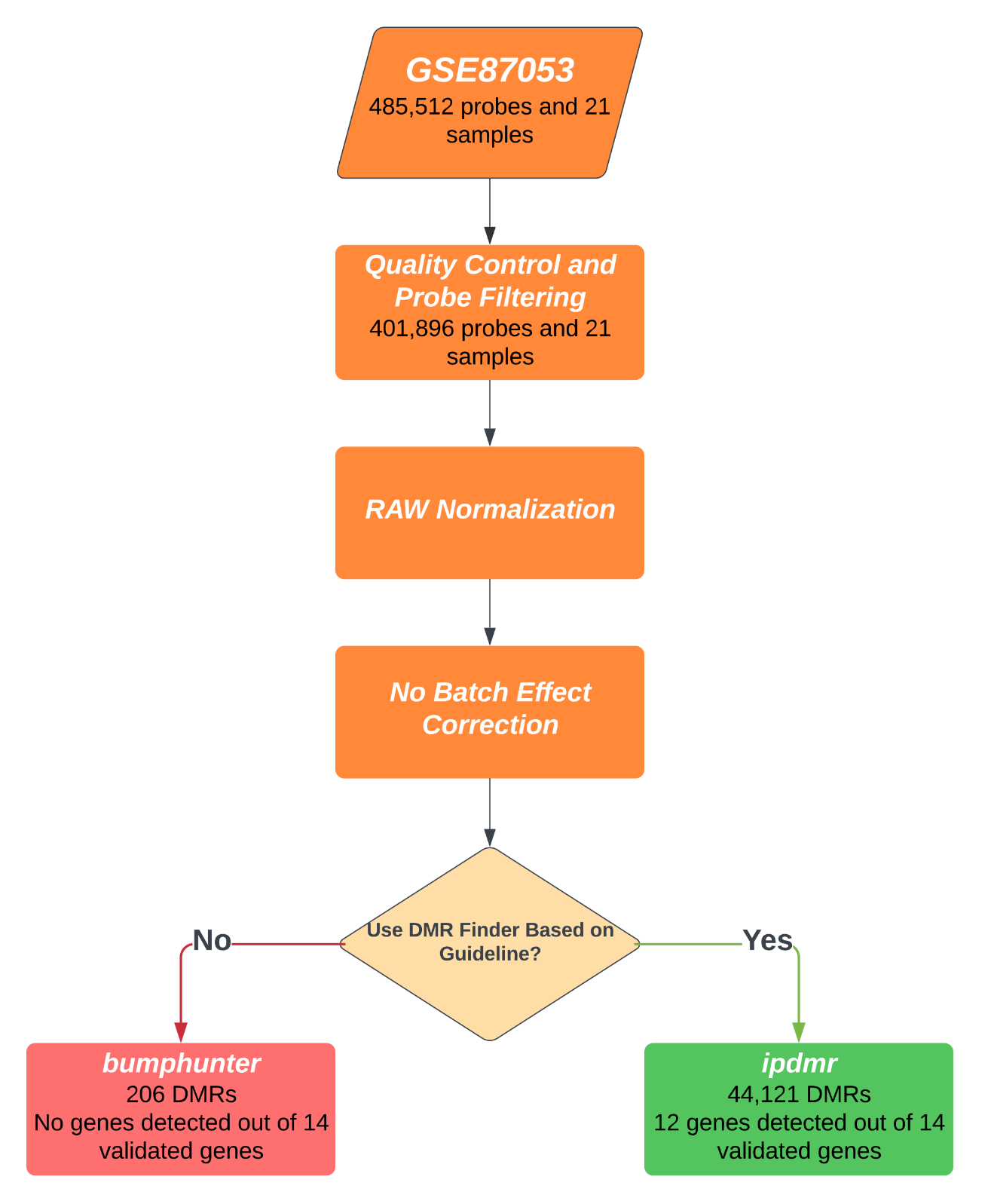
**

*The outcomes derived from running the proposed workflow and its validation. The workflow was applied to an independent dataset to confirm its ability to identify the reported and laboratory-validated genes (results in green box). Simultaneously, an alternative workflow was executed for performance comparison (results in red box). The findings show a significantly enhanced performance of the proposed workflow.*

**Supplementary Tables’ Legend**

Supplementary Table 1 - Comparison of different normalization techniques (BMIQ, betaQN, BAQN) on all simulation scenarios. To compare, four evaluation metrics were used: 1- Median of probe SDs (Standard Deviations) across samples, 2- Median of type-1 probe SDs across samples, 3- Median of type-2 probe SDs across samples, and 4- dmrse (differentially methylated region standard error). BAQN outperformed other techniques in all simulation scenarios.

Supplementary Table 2 - Results from evaluating different pipelines in analyzing all simulation scenarios. The tables are the evaluation metrics (precision, recall, accuracy, and F1-score) calculated to assess the DMP finding pipelines in all 12 scenarios.

Supplementary Table 3 - Results from evaluating different pipelines in analyzing all simulation scenarios. The tables are the evaluation metrics (precision, recall, accuracy, and F1-score) calculated to assess the DMR finding pipelines in all 12 scenarios.

Supplementary Table 4 - Results from evaluating different pipelines in analyzing all simulation scenarios. The tables are the evaluation metrics (precision, recall, accuracy, and F1-score) calculated to assess the DMR finding pipelines in detecting regions with small difference in the two groups.

Supplementary Table 5 - Results from evaluating different pipelines in analyzing all simulation scenarios. The tables are the evaluation metrics (precision, recall, accuracy, and F1-score) calculated to assess the DMR finding pipelines in detecting regions with large difference in the two groups.
